# CD-HIT: accelerated for clustering the next-generation sequencing data

**DOI:** 10.1093/bioinformatics/bts565

**Published:** 2012-10-11

**Authors:** Limin Fu, Beifang Niu, Zhengwei Zhu, Sitao Wu, Weizhong Li

**Affiliations:** Center for Research in Biological Systems, University of California San Diego, La Jolla, CA 92093, USA

## Abstract

**Summary:** CD-HIT is a widely used program for clustering biological sequences to reduce sequence redundancy and improve the performance of other sequence analyses. In response to the rapid increase in the amount of sequencing data produced by the next-generation sequencing technologies, we have developed a new CD-HIT program accelerated with a novel parallelization strategy and some other techniques to allow efficient clustering of such datasets. Our tests demonstrated very good speedup derived from the parallelization for up to ∼24 cores and a quasi-linear speedup for up to ∼8 cores. The enhanced CD-HIT is capable of handling very large datasets in much shorter time than previous versions.

**Availability:** http://cd-hit.org.

**Contact:** liwz@sdsc.edu

**Supplementary information:** Supplementary data are available at *Bioinformatics* online.

## 1 INTRODUCTION

Sequence analysis has played a crucial role in computational biology. With the advancement of the next-generation sequencing technologies, the amount of available sequencing data is growing exponentially. Removing redundancy from such data by clustering could be crucial for reducing storage space, computational time and noise interference in some analysis methods, etc.

CD-HIT was originally developed to cluster protein sequences to create reference databases with reduced redundancy (Li *et al.*, 2001) and was then extended to support clustering nucleotide sequences (Li and Godzik, 2006). Since its release, CD-HIT has become very widely used for a large variety of applications ranging from non-redundant dataset creation (Suzek *et al.*, 2007), protein family classifications (Yooseph *et al.*, 2008), artifact identification (Niu *et al.*, 2010), metagenomics annotation (Sun *et al.*, 2011), RNA analysis (Loong and Mishra, 2007), to various prediction studies (Rubinstein and Fiser, 2008).

With sequencing data rapidly growing in public data repositories as well as in individual laboratories, there has been strong demand for an enhanced CD-HIT with greater efficiency. In response to such demand, we have developed this enhanced and parallelized version of CD-HIT, to exploit the fact that multi-core machines have become very common in ordinary laboratories.

A computer cluster-based parallelization procedure for CD-HIT has been proposed in Suzek *et al.* (2007), though not fully parallelized, this procedure provides good speedup using computer cluster. Since computer clusters are not as easily available as multi-core machines, here we propose an alternative parallelization technique, which assumes shared memory model and works well on multi-core machines.

## 2 METHODS

Basically, CD-HIT is a greedy incremental algorithm that starts with the longest input sequence as the first cluster representative, and then process the remaining sequences from long to short to classify each sequence as a redundant or representative sequence based on its similarities to the existing representatives. The similarities are estimated by common word counting using word indexing and counting tables to filter out unnecessary sequence alignments, which are used to compute exact similarities. In the following sections, we will describe the techniques that are used to accelerate CD-HIT.

### 2.1 Simplification

In order to support full parallelization, the core steps of CD-HIT have been simplified into two key procedures: a checking procedure and a clustering procedure. Using these two procedures, the algorithm requires at most two word tables without the need to swap them to disk, which was necessary in the original CD-HIT due to the use of multiple tables for large datasets.

Given a word table, the checking procedure checks each of the remaining sequences against the table and determines whether it is a redundant sequence. The clustering procedure will make a final determination of the status of a sequence, and if it is classified as a representative sequence, it is used to update the word table. A more detailed description with illustration is available in Supplementary Material Section 1.2 and Figure S1.

### 2.2 Parallelization

Given *T* threads or cores, the basic idea of our novel parallelization technique is to use two word tables and use *T*−1 threads to run multiple checking procedures using one word table (a immutable checking table), and the remaining thread to run a single clustering procedure using the other table (a mutable clustering table) in parallel. Due to the sequential characteristics of CD-HIT, it will require properly grouping the input sequences and switch the word tables to guarantee the correctness of the parallelization.

This is achieved by dividing each round of computation into two stages. The first stage is to run *T* checking procedures on the sequence group defined for this round of computation using the word table (checking table) from the previous round. Then, the second stage will use an additional and empty word table (clustering table) to run a clustering procedure in one thread on the current sequence group, and at the same time the remaining *T*−1 threads will run multiple checking procedures on the remaining sequences.

Since the clustering procedure may finish before or after the checking procedures, proper scheduling is used to guarantee all threads are active most of the time. At the end of each round, the clustering table will become the checking table of the next round, and the checking table of this round will be emptied to become the clustering table of the next round. More information including detailed description, illustration and pseudo codes, etc. are available in the Supplementary Material (Sections 1.3–1.5).

### 2.3 Other enhancements

Besides the parallelization, the new CD-HIT includes other enhancements such as faster file reading, better filtering threshold estimation, more efficient word counting and better alignment band estimation, etc. The new filtering threshold estimation is slightly more precise and can filter out more unnecessary alignments. Now word counting is handled more efficiently for input datasets with high redundancy, by maintaining a smaller counting array for hit representatives instead of a full counting array for every representatives. The improved alignment band estimation can find a narrower band for banded alignment.

### 2.4 Implementation

This enhanced CD-HIT is implemented in the C++ programming language and uses OpenMP (http://www.openmp.org) for parallelization. The parallel for construct of OpenMP is used for running the checking and clustering procedures in multiple threads with dynamic scheduling. Different computation data buffers are allocated for different threads. The checking word table is immutable and shared by multiple threads.

## 3 RESULTS

To see how much performance improvement has been achieved, we tested the new CD-HIT (V4.6) and the old CD-HIT (V3.1.2) on a set of datasets including two protein sequence datasets: SWISS-PROT (∼0.4 M sequences), NR (∼12 M sequences) and two DNA sequence datasets: HumanGut (MH0002, ∼23 M reads; Qin *et al.*, 2010) and TwinStudy (SRP000319, ∼8 M reads; Turnbaugh *et al.*, 2009). Both the SWISS-PROT and the NR datasets were downloaded from NCBI (ftp://ftp.ncbi.nih.gov/blast/db/FASTA/) on October 20, 2010. We also compared CD-HIT with a similar program UCLUST (V5.1.221) from Edgar (2010). These tests were done on a Debian Linux server with four 12-core AMD Opteron 6172 processors. Equivalent parameters were used for different programs whenever possible. Details and additional tests are available in Supplementary Material 2.

**Fig. 1.**
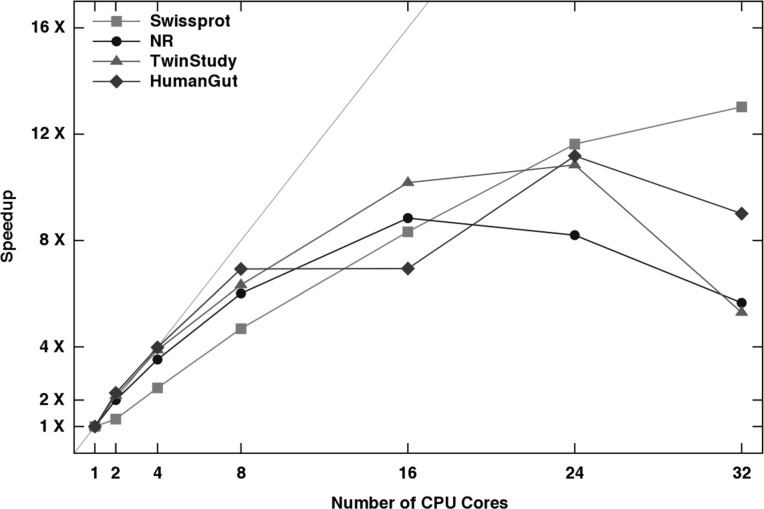
Evaluation of CD-HIT parallelization: computational time speedup with respect to the number of used CPU cores

Table 1 compares the efficiency of the enhanced CD-HIT to the previous version of CD-HIT and the latest UCLUST. The results demonstrate that the new CD-HIT without using multi-core is significantly more efficient than the old one and is comparable to or more efficient than UCLUST as well. When multi-cores are used, the new CD-HIT is much more efficient than either of them. To test the effectiveness of the parallelization in the new CD-HIT, the full datasets were clustered using different number of cores. The time speedups are shown in Figure 1, which indicates the parallelization has good speedup for up to ∼24 cores with a quasi-linear speedup for up to ∼8 cores. Besides speed improvements, the new CD-HIT also has better clustering quality than the old CD-HIT and UCLUST (Supplementary Material and Table S2).

**Table 1.** Comparison to the previous CD-HIT and UCLUST

Dataset	CD-HIT3 (min)	CD-HIT4 (min)	CD-HIT4 (8 cores) (min)	UCLUST5 (min)
Swissprot	80	58	12	15
NR	44	22	6	46
Twinstudy	47	19	4	56
HumanGut	494	42	8	214

UCLUST5 free version cannot run on the full NR, TwinStudy and HumanGut datasets, so subsets with ∼1 M sequences of NR, 1 M reads of TwinStudy and 4 M reads of HumanGut are used in this comparison.

## 4 CONCLUSIONS

In this application note, we presented an enhanced CD-HIT that has been accelerated by a novel parallelization technique and a few other improvements. We tested and demonstrated that this new CD-HIT can achieve significant speedup over the previous CD-HIT using a single core, and its acceleration by multi-core computer can scale up well to a reasonable number of cores. Clustering on large datasets that normally runs for days can now finish in hours on multicore machines. We believe this enhanced CD-HIT will find more applications in handling the next-generation sequencing data.

*Funding*: This study was supported by National Institute of Health award R01RR025030 from the National Center for Research Resources and award R01HG005978 from the National Human Genome Research Institute.

*Conflict of Interest*: none declared.

## Supplementary Material

Supplementary Data

## References

[bts565-B1] Edgar RC (2010). Search and clustering orders of magnitude faster than BLAST. Bioinformatics.

[bts565-B2] Li W, Godzik A (2006). Cd-hit: a fast program for clustering and comparing large sets of protein or nucleotide sequences. Bioinformatics.

[bts565-B3] Li W (2001). Clustering of highly homologous sequences to reduce the size of large protein databases. Bioinformatics.

[bts565-B4] Loong SNK, Mishra SK (2007). Unique folding of precursor microRNAs: quantitative evidence and implications for de novo identification. RNA.

[bts565-B5] Niu B (2010). Artificial and natural duplicates in pyrosequencing reads of metagenomic data. BMC Bioinformatics.

[bts565-B6] Qin J (2010). A human gut microbial gene catalogue established by metagenomic sequencing. Nature.

[bts565-B7] Rubinstein R, Fiser A (2008). Predicting disulfide bond connectivity in proteins by correlated mutations analysis. Bioinformatics.

[bts565-B8] Sun S (2011). Community cyberinfrastructure for Advanced Microbial Ecology Research and Analysis: the CAMERA resource. Nucleic Acids Res..

[bts565-B9] Suzek BE (2007). UniRef: comprehensive and non-redundant UniProt reference clusters. Bioinformatics.

[bts565-B10] Turnbaugh PJ (2009). A core gut microbiome in obese and lean twins. Nature.

[bts565-B11] Yooseph S (2008). Gene identification and protein classification in microbial metagenomic sequence data via incremental clustering. BMC Bioinformatics.

