# Supplementary material for: CD-HIT: accelerated for clustering the next-generation sequencing data
Source: Bioinformatics. 2012 Oct 11;28(23):3150–2. doi: 10.1093/bioinformatics/bts565 (PMC3516142; doi:10.1093/bioinformatics/bts565)
Supplement: Supplementary Data [file supp_28_23_3150__index.html]

CD-HIT: accelerated for clustering the next-generation sequencing data — Supplementary Data 

# CD-HIT: accelerated for clustering the next-generation sequencing data

## Supplementary Data

files

**Files in this Data Supplement:**

- Supplementary Data - doc file
