# Supplementary material for: CD-HIT: accelerated for clustering the next-generation sequencing data
Source: Bioinformatics. 2012 Oct 11;28(23):3150–2. doi: 10.1093/bioinformatics/bts565 (PMC3516142; doi:10.1093/bioinformatics/bts565)
Supplement: Supplementary Data [file supp_bts565_bts565.doc]

**Supplementary materials**

**CD-HIT: accelerated for clustering the next generation sequencing data**

Limin Fu, Beifang Niu, Zhengwei Zhu†, Sitao Wu and Weizhong Li*

Center for Research in Biological Systems, University of California San Diego, La Jolla, California, United States of America

* To whom correspondence should be addressed ([liwz@sdsc.edu](mailto:liwz@sdsc.edu)).

# Algorithm Description and Illustration

## Basic CD-HIT Algorithm

The basic CD-HIT algorithm sorts the input sequences from long to short, and processes them sequentially from the longest to the shortest. The first sequence is automatically classified as the first cluster representative sequence. Then each query sequence of the remaining sequences is compared to the representative sequences found before it, and is classified as redundant or representative based on whether it is similar to one of the existing representative sequences.

Such comparison is done in an efficient way by using word counting and indexing table to filter out most of the unnecessary comparisons by a filtering process. For each word from the query sequence, this process retrieves a list of representative sequences that contain the word hits using the word table, and in the mean time, the count of common words between the query and each of the retrieved representatives is updated. Then only those representatives with common word counts above certain threshold are passed to an alignment procedure to compute the similarity (alignment identity) between the query and the representative. The alignment procedure also uses word-counting techniques to define a proper band to do efficient banded alignment. If the alignment identity satisfies the specified requirement, the query sequence is classified as a representative sequence, and the word table is subsequently updated to include the counts of the words found in this new representative sequence.

## Basic CD-HIT Algorithm with Limited Table Size

When a huge number of sequences are classified as representatives, the word table will become very large and require a massive amount of memory. In the original implementation, the input sequences are divided into groups (segments), which are processed one after another. One word table is generated from the representative sequences of each processed group. The sequence data of each processed group and the associated word table are swapped into the memory from the disk when needed, and swapped out otherwise, to reduce memory usage.

In the new CD-HIT, we redesigned the memory reduction strategy to remove the need for swapping word tables between memory and disk. The idea is to maintain only one active sequence group and construct one word table from the representative sequences obtained by clustering the sequences in the active group. Then the constructed word table is used to process all the remaining sequences so that the sequence group and word table can be discarded or cleared for handling the next sequence group. This strategy of sequence grouping and size-constrained word table construction forms the basics of our parallelized CD-HIT algorithm that will be described in the next sub-section.

To better illustrate the clustering process with grouped sequences, we define two types of procedures: *checking* and *clustering*. Checking is the procedure to check a sequence against a word table to determine if the sequence is redundant. Clustering is the procedure to determine if a sequence is a representative sequence, and if yes, update the word table with the words from this new representative sequence. For the correctness of the algorithm, each sequence has to be checked against the word tables for the previous groups before it can be clustered against the word table of the current group.


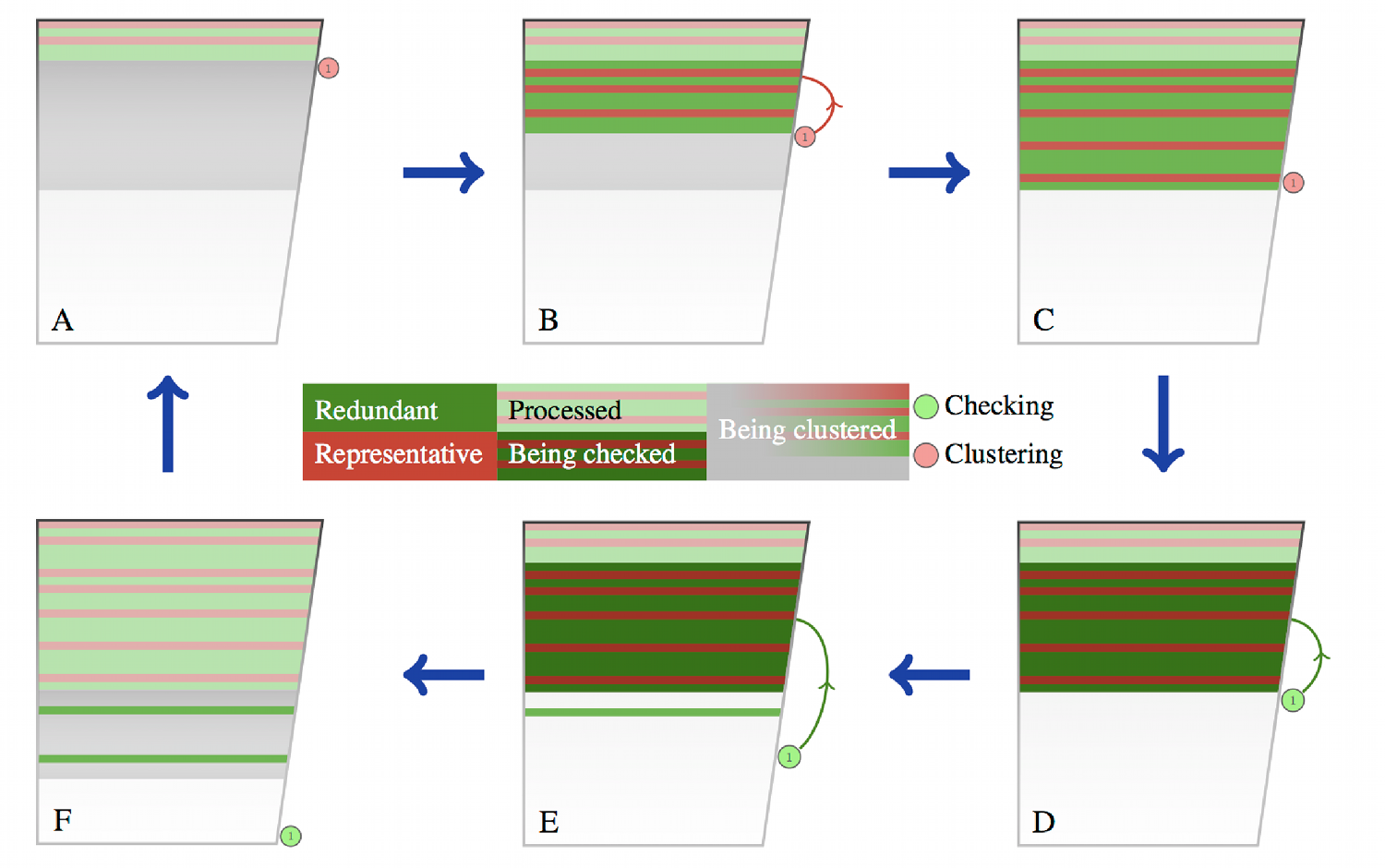


**Figure S1.** **Basic CD-HIT algorithm with size-constrained word table**

Representative and redundant sequences are marked as red and green respectively. The blocks with light red and green stripes **(A-F)** represent processed sequences that have been clustered and are no longer needed to process other sequences. The gray blocks are the current group of sequences to be processed by the clustering procedure. The dark red and green strips are the sequences being used to check the rest of the sequences. **(A)** each new round of clustering procedure starts with the first gray sequence and an empty word table; **(B)** the clustering procedure will mark new redundant sequences and update the word table with new representative sequences; **(C)** the clustering procedure is carried out until all the gray sequences in the current group are processed; **(D)** then the checking procedure starts with the first unmarked sequence; **(E)** the checking procedure will mark new redundant sequences; **(F)** the clustering procedure is carried out until all the remaining unmarked sequences are processed; then a new sequence group is defined for the next round of clustering and checking procedures starting from **A**.

So the new basic clustering algorithm looks like the following: the first sequence is clustered as a representative by default and is used to build the word table. The following sequences are clustered against this table one by one, until the size of the table reaches the predefined limit. Then each of the remaining sequences in the dataset is checked against the table and marked as redundant if necessary. Those marked as redundant will be skipped in the following procedures. After all the sequences have been checked, we clear the word table and repeat the same steps of clustering and checking procedures, starting from the first sequence that has not gone through the clustering procedure. The algorithm stops when all the sequences have been either marked as redundant or processed by the clustering procedure. Figure S1 illustrates the key steps of each clustering and checking cycle in the basic CD-HIT algorithm.

## Parallelization Strategy

The basic algorithm needs only one word table throughout the whole clustering process. But to realize an effective parallelization of the clustering process, we need two word tables, one will be called checking table, the other clustering table. The usage of these two different tables will become clear in the following description of the parallelization technique.

To reduce unparallelized execution, the first group is usually small, and can be processed by the original sequential CD-HIT algorithm to cluster those sequences and build the first word table. Of course, the first group can also be subdivided into even smaller groups, and parallelized within them.

Once we have the first word table, we will take this table as the checking table and create an empty clustering table. Now the following computation can be done in parallel as a two-stage process, which is repeated until finish. The first stage will carry out multiple checking procedures in parallel. For this, we define a new group of sequences (lighter gray section in Figure S2 A, B, C), and use multiple threads (green nodes and arrows on the right side of the sub-figures) to check the sequences of this group against the current checking table. Once this stage is done, the second stage will carry out one clustering procedure/thread and one or multiple checking procedure(s)/threads(s) in parallel as the following: the clustering procedure (red node and arrow on the right side of Figure S2 D, E, F) will be carried out on the new group of sequences that have just finished the checking procedures in the first stage; and the checking procedure(s) will check the remaining sequences against the current checking table. The clustering procedure will use and update the clustering table.

The second stage will proceed until all the remaining sequences have been checked against the current checking table. If the clustering thread has finished processing the sequences in the new group, it will switch to do the checking procedure. And if the checking threads finished before the clustering thread, the clustering thread will be terminated to finish the current cycle of parallelization. This will guarantee that all the threads will be doing active computation. Once the second stage is done, the clustering table will become the current checking table, and the previous checking table will be cleared and used as the current clustering table. Then these two stages of computation will be repeated starting from the first sequence that hasn't been processed by the clustering procedure.


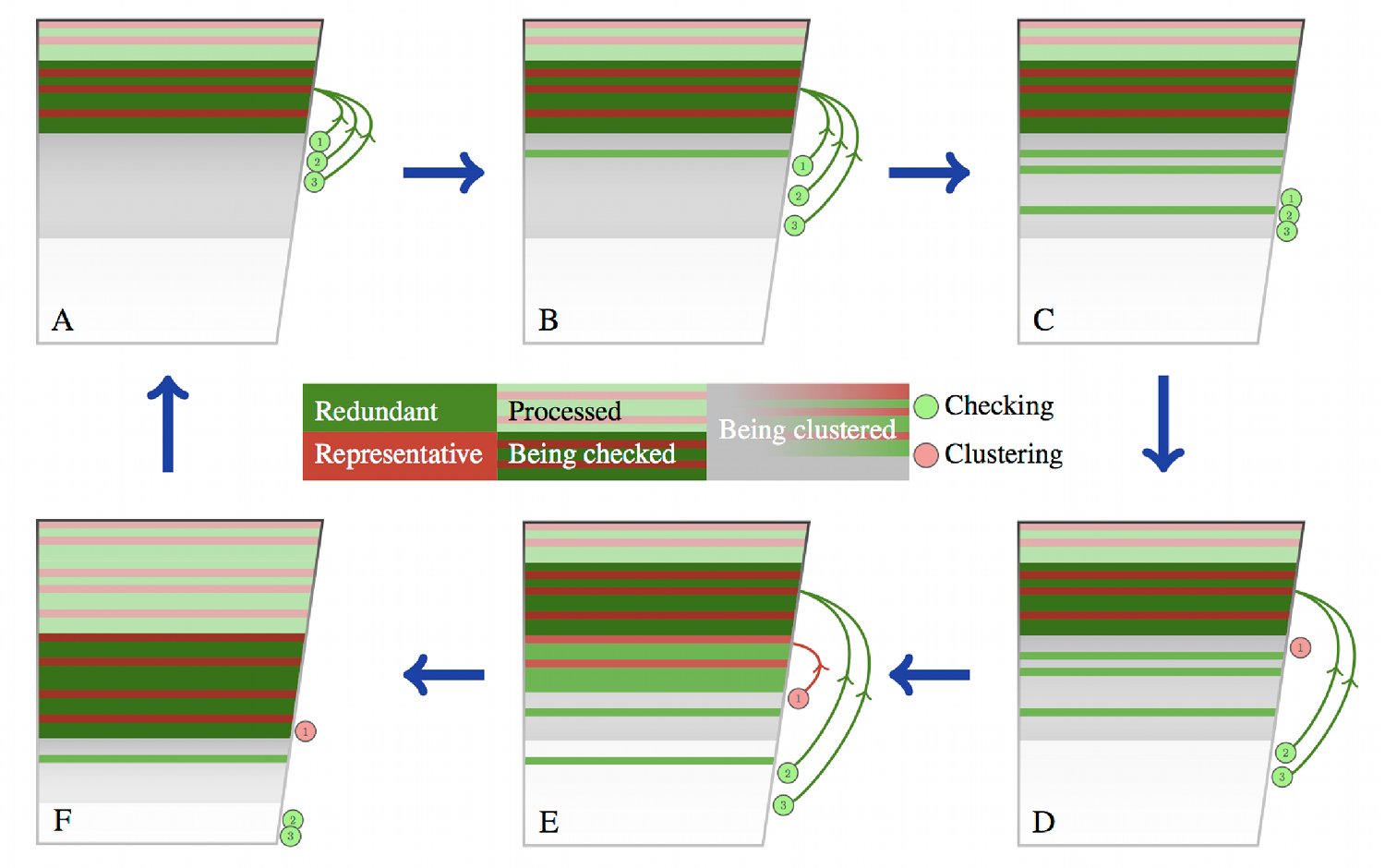


**Figure S2.** **Parallelized CD-HIT algorithm**

The color denotations in this figure are identical to those in **Figure S1**. Here the light green and red circles stand for checking threads and clustering threads respectively. The sequence of steps **A-F** has been rotated with respect to that in **Figure S1** to simplify the illustration of the parallel algorithm. Before starting the first round of step **A**, it is assumed that a small group of sequences have been already processed by the sequential CD-HIT algorithm to build the first word table that will be used as the checking table in **A**. **(A)** redefine the current group of sequences, use all threads to run checking procedures on the current group in parallel; **(B)** each thread takes one unmarked sequence and check it against the checking table to determine if it is a redundant sequence; **(C)** the checking procedures are carried out until all the sequences in the current group have been processed; **(D)** the first (clustering) thread runs the clustering procedure starting from the beginning of the current sequence group; in the mean time, the other (checking) threads run the checking procedures on the remaining sequences in the dataset; **(E)** the clustering thread detects new representative sequences and uses them to update the clustering table; the checking threads detect and mark new redundant sequences; **(F)** the clustering and checking procedures are continued until the checking procedures reaches the end of the dataset. The algorithm repeats A through F until all sequences have undergone the clustering procedure.

## Pseudo Codes

To better illustrate the parallelization algorithm, we supplement the text description and figures with the following pseudo codes. In the codes, a *PARALLEL FOR* statement means multiple threads can execute the loop body in parallel with the index variable taking a different value. Here we assume each thread has a private copy of the index variable, and is dynamically scheduled to take the first index value that hasn't been processed by any thread. Different computation data buffers should be allocated for different threads, and the checking word table is immutable and can be shared by multiple threads. The parallelization model proposed here is very suitable to be implemented with OpenMP for multi-core machines.

**-- define a group starting from sequence index "first":**

**-- return the index of the last sequence in the group:**

**PROCEDURE DefineGroup( first );**

**-- use "table" to check "sequence":**

**-- mark "sequence" if it is redundant:**

**PROCEDURE Checking( table, sequence );**

**-- use "table" to cluster "sequence":**

**-- mark "sequence" as redundant or representative:**

**-- update "table" if "sequence" is a representative:**

**PROCEDURE Clustering( table, sequence );**

**PROCEDURE ParallelCDHIT( sequences, N )**

**-- create table for checking procedures**

**tabcheck = NEW table;**

**-- create table for the clustering procedure**

**tabclust = NEW table;**

**first = 1;**

**-- all loops can skip sequences marked as redundant**

**WHILE first <= N DO**

**last = DefineGroup( first );**

**IF tabcheck NOT empty THEN**

**-- first stage:**

**PARALLEL FOR j = first...last DO**

**-- all threads do the checking procedure**

**Checking( tabcheck, sequences[j] );**

**END**

**checked = FALSE;**

**-- second stage:**

**PARALLEL FOR j = last...N DO**

**IF j == last THEN**

**-- the first thread does clustering**

**FOR k = first...last DO**

**Clustering( tabclust, sequences[k] );**

**IF checked == TRUE THEN**

**first = k + 1;**

**BREAK;**

**END**

**END**

**ELSE**

**-- all other threads do checking**

**Checking( tabcheck, sequences[j] );**

**IF j == N THEN checked = TRUE; END**

**END**

**END**

**END**

**IF first == 1 OR last == N THEN**

**-- handle the first and last group**

**FOR k = first...last DO**

**Clustering( tabclust, sequences[k] );**

**END**

**END**

**-- clustering table becomes checking table**

**SWAP tabcheck, tabclust;**

**CLEAR tabclust;**

**END**

**END**

## Analysis of The CD-HIT Parallelization Algorithm

This parallelization algorithm is very effective for large datasets. In fact, for large datasets, the time spent in handling the first group and the last group can be neglected, hence the effectiveness or the speed-up of the parallelization is mostly determined by the two parallel-for loops in the pseudo codes. But it is clear that the first parallel loop can be perfectly parallelized because each of the sequences in the group can be checked independently against the checking table. So the speed-up will be mostly affected by the second parallel loop.

The situation in the second parallel loop is a bit more complicated, because not all the threads do the same type of computation. Due to the dynamic scheduling of the threads, the first thread will start with the first index value and execute the inner loop to do the clustering procedure, while the other threads will loop on the other index values to do the checking procedures. Now it may happen that it takes longer time for the inner loop to finish than the outer loop to reach its last index. When this happens, the inner loop will be interrupted to ensure the thread running the inner loop will not become the only active thread. When the inner loop is interrupted, the algorithm will start the next round of the two stages of parallel computation starting with the index where it left the inner loop. But if the inner loop finishes before the outer loop reaches the last index, the thread running the inner loop will switch to do checking procedures as well. In this case, all the threads are still all active.

So in the ideal case, all the threads of the two stages of parallel computation will be active. If the dataset is big enough, the time spent in handling the first and the last group will be negligible, so the speedup can theoretically approach the number of used CPU cores. However, the sizes of the checking table and clustering table are determined by the size of the input dataset, the memory limit specified for the program and the number of used CPU cores. When sequences are short and the table sizes become small, the overhead of thread context switching due to the multi-cycle of two-stage parallel computation will become significant enough to reduce the speedup of the parallelization.

# Detailed Testing Results

The datasets used for testing are described in the main manuscript.

## Comparisons to The Previous CD-HIT and UCLUST

The results (Table S1) indicate the new CD-HIT, even without parallelization, is significantly faster than the old one in most cases, and produces fewer clusters with the same identity thresholds, which means the filtering by word table and the sequence similarity assessment in the new CD-HIT is better than that in the old one. These results did not confirm the original claim that UCLUST is superior to CD-HIT in terms of speed (Edgar, 2010). In fact, only in the test with low identity, UCLUST is faster than CD-HIT, while in the other cases it is significantly slower than CD-HIT.

**Table S1.** Comparison to the previous CD-HIT and UCLUST


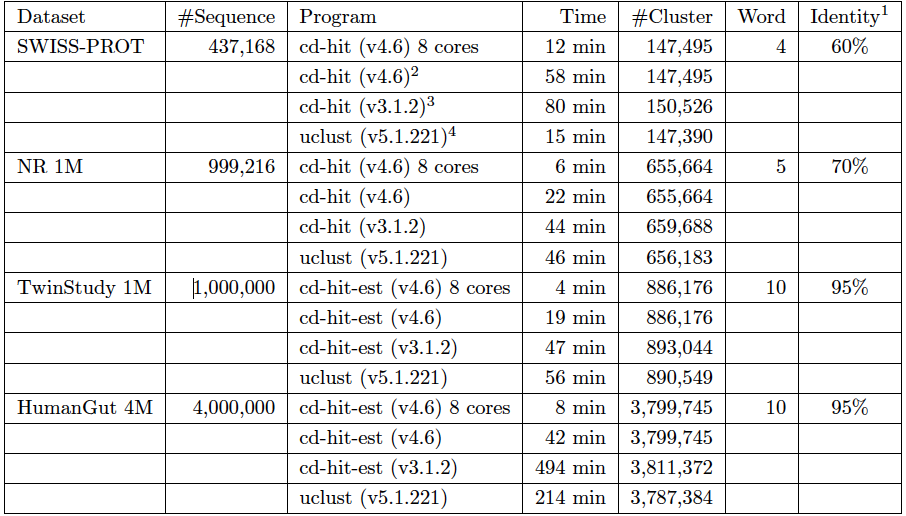


1. All these programs take a fraction identity as the identity cuto_ parameter, namely, 0.6 is used for 60% and 0.7 for 70% etc.;

2. CD-HIT 4.6 command line: program -i dataset -o output -c identity -n word -M 0 -T nthread. Number of threads nthread is 1 for tests using one core and 8 for 8 cores; For DNA sequence dataset, -r 0 is used to avoid checking reverse complementaries for sequence similarities;

3. CD-HIT 3.1.2 command line: program -i dataset -o output -c identity -n word -M max memory. Maximum memory max memory is 400 and 1000 (MB) for the SWISS-PROT and HumanGut datasets, and 5000 (MB) for the other two;

4. UCLUST 5.1.221 command line: uclust --cluster dataset --seedout output --id identity --w word --k sword --band 20 --maxlen 1000000 --minlen 10. Seed word length --k sword takes the default values (3 for amino acids and 4 for nucleotides).

Since there is no golden standard datasets for testing the biological accuracy of such sequnence clustering programs, we use BLAST to evaluate the algorithmic accuracy of these programs. Because different programs use different heuristics to improve the speeds, some redundant sequences will be missed and clustered as representative (non-redundant) sequences, so the representatives produced by each of the programs may contain a different amount and small fraction of redundant sequences which can be identified by BLAST.

For each set of representatives, we define the redundancy ratio as the number of missed redundant sequences divided by the total number of representative sequences, and use it as a measure of program's clustering accuracy. For example, after clustering by a program (e.g. CD-HIT 4) at certain identity (e.g. 60%), we run all-by-all BLAST (BLASTP or BLASTN) search on the non-redundant sequence produced by CD-HIT 4 and identify redundant sequences at 60% by blast alignments, which are the missed redundant sequences by CD-HIT 4. The redundancy ratio is missed redundant sequences / total representative sequences. The redundancy ratios of the cluster representatives produced by the three programs are shown in the Table S2. Due to the computational time of all-by-all BLAST searching, only subsets (the first 50K) of the cluster representatives of the small datasets are evaluated.

**Table S2.** Redundancy ratios of cluster representatives


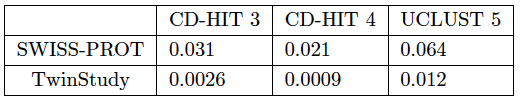


**Table S3.** Tests of the enhanced CD-HIT on the full-sized datasets


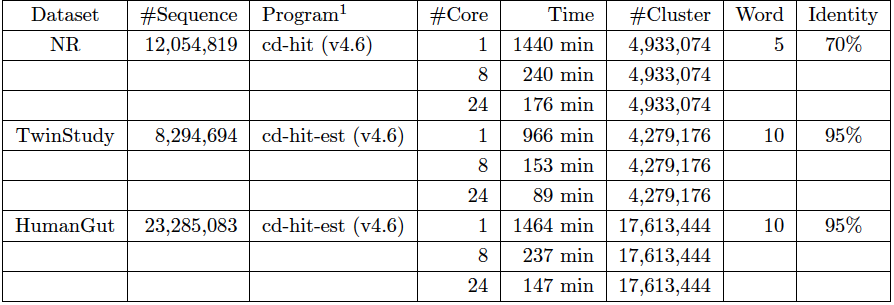


1. The parameters used in this table are the same as those used in the Table S1 for CD-HIT 4.6, except that cd-hit-est uses option -r 1 to check the reverse complementary for sequence similarities.

## Parallelization Speedup and Scalability Against The Number of CPU Cores

To assess the effectiveness (speedup) of the proposed parallelization technique, we tested the new CD-HIT with different number (1, 2, 4, 8, 16, 24 and 32) of threads on the same set of datasets (full sized version). The run time of tests with 1, 8 and 24 threads on the three large full sized datasets are listed in Table S3. The results (Figure S3, left) demonstrate that the parallelization speedup scales well with up to 24 CPU cores, before it starts to degrade. The parallelization performs particularly well with 8 and less number of cores, in fact, for the big datasets, the speedups are almost linear and approach the number of used cores. Before the parallelization starts to degrade for large number of cores, it is also clear that higher speedup is achieved on bigger datasets with few exceptions. There are a few factors that could have possibly contributed to such performance degradation, such as context switching for multiple threading, limited cache size and memory bandwidth etc. Since the speedup did not degrade on the smallest dataset (SWISS-PROT) for using 32 cores, the degradation is more likely due to the limited cache size and memory bandwidth. When the number of cores increases, the immutable shared word table will be accessed more frequently, which will result in more cache misses and more main memory accessing for large word tables. Main memory access is much slower than cache access due to latency and limited bandwidth, with increasing number of cores, at certain point, it will become a bottleneck for the speed. The tests on the SWISS-PROT dataset used smaller word size and a lower identity cutoff, which leaded to small word tables, so that the cache size and memory bandwidth limit had none or little impact on the speed when more cores are used.


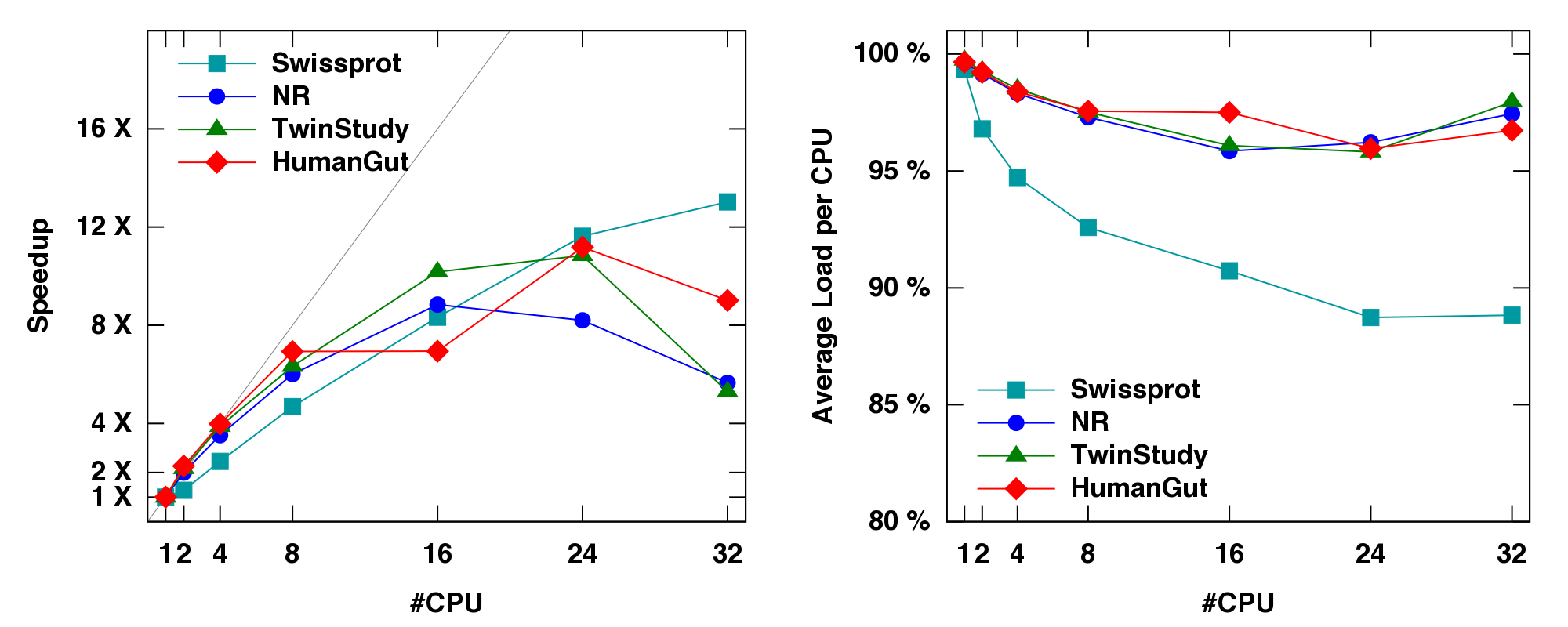


**Figure S3.** **Evaluation of the parallelized CD-HIT:** Parallelization scalability against the number CPU cores. Left: computational time speedup with respect to the number of used CPUs. Right: average CPU load as the percentage CPU usage per thread.

The effectiveness is also evaluated by the average load per CPU in the parallelization. The average load per CPU is computed as the ratio between the total CPU time of all used CPUs and the execution time multiplied by the number of CPUs. The results (Figure S3, right) indicate that the load per CPU is generally high and drops slightly when the number of CPUs increases. And as expected, the CPU load is generally higher for bigger datasets than for smaller datasets.

## Parallelization Speedup and Scalability Against Dataset Sizes

To see how the parallelization scales with the size of datasets, we created subsets of the datasets with approximately 1, 2, 4, 6 and 8 million sequences, and then run CD-HIT on them without parallelization and with parallelization using 8 cores. Some of the tests have been run multiple times, but only small variations are observed in the speeds, so we only present the results of one run per test. %Here each test is also performed 3 times on different computer nodes (but of the same type). The speedups and average CPU loads are shown in Figure S4. The left figure shows the speedups increase with the sizes of input datasets increase, until the best speedups are achieved at around 6 million sequences. %The speedup degradation for 8 millions and up sequences, it might be the consequence %of limited cache capacity and memory bandwidth for increased word table sizes. While for CPU loads, there is an apparent trend in which the load per CPU increases when the input dataset size increases.


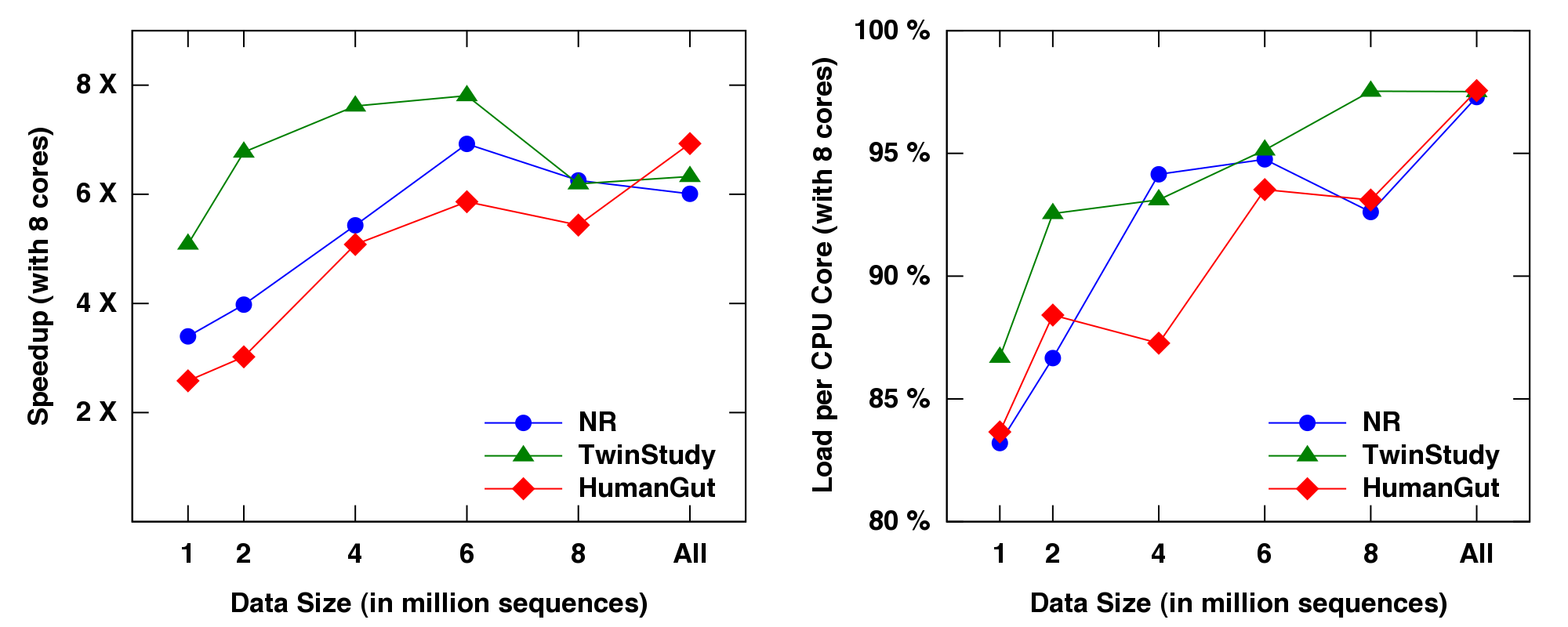


**Figure S4.** **Evaluation of the parallelized CD-HIT:** parallelization scalability against dataset sizes. Left: computational time speedup with respect to the input dataset size. Right: average CPU load as the percentage CPU usage per thread.
